# Invasive Aspergillosis Involving the Mediastinum in an Immunocompetent Patient: A Case Report

**DOI:** 10.7759/cureus.1605

**Published:** 2017-08-24

**Authors:** Manesh Kumar Gangwani, Muhammad Aziz, Siraj Munir, Syed Ahsan Ali

**Affiliations:** 1 Medical College, Aga Khan University Hospital, Karachi; 2 Department of Medicine, Kansas University Medical Center, Kansas City, KS.; 3 Department of Medicine, Aga Khan University Hospital, Karachi

**Keywords:** invasive pulmonary aspergillosis, immunocompetence, infective endocarditis

## Abstract

We report a rare case of invasive pulmonary aspergillosis invading the mediastinum and the left atrium. A 38-year-old female was hospitalized for cough, shortness of breath and fever. She had a past medical history of tuberculosis. Computed tomography (CT) scans identified an ill-defined enhancing mediastinal soft tissue density mass encasing the heart and major vessels. The cardiac echocardiography showed global hypokinesia, low ejection fraction and a large echogenic density in the left atrium. The pathology from the bronchoscopic biopsy observed abundant fungal hyphae which were stained with periodic Acid-Schiff and Gomori's methenamine silver. Despite the treatment with antifungal agents, the patient could not be saved. Invasive pulmonary aspergillosis, which involves the mediastinum and the heart, is very rare in immunocompetent patients.

## Introduction

Invasive aspergillosis can be found in an immunocompetent host, however, it is unusual and a significantly serious complication in immunocompetent hosts [1]. The lungs are predominantly involved; however, it could potentially involve various organs as reported in the literature before [2]. A low index of suspicion and its rarity make it a very challenging diagnosis. Here we present a case of a middle age immunocompetent female with biopsy proven invasive aspergillus mediastinal mass causing fungal invasive endocarditis, which led to her mortality. Informed consent was obtained from the patient for this study

## Case presentation

A 38-year-old South-Asian female was admitted with worsening cough, shortness of breath and fever for three days. She had these symptoms for over one year. She was afebrile at the time of examination and her vitals were normal and stable. On physical examination, chest veins were prominent throughout and on inspection, the auscultation was notable for stridor.

**Figure 1:**
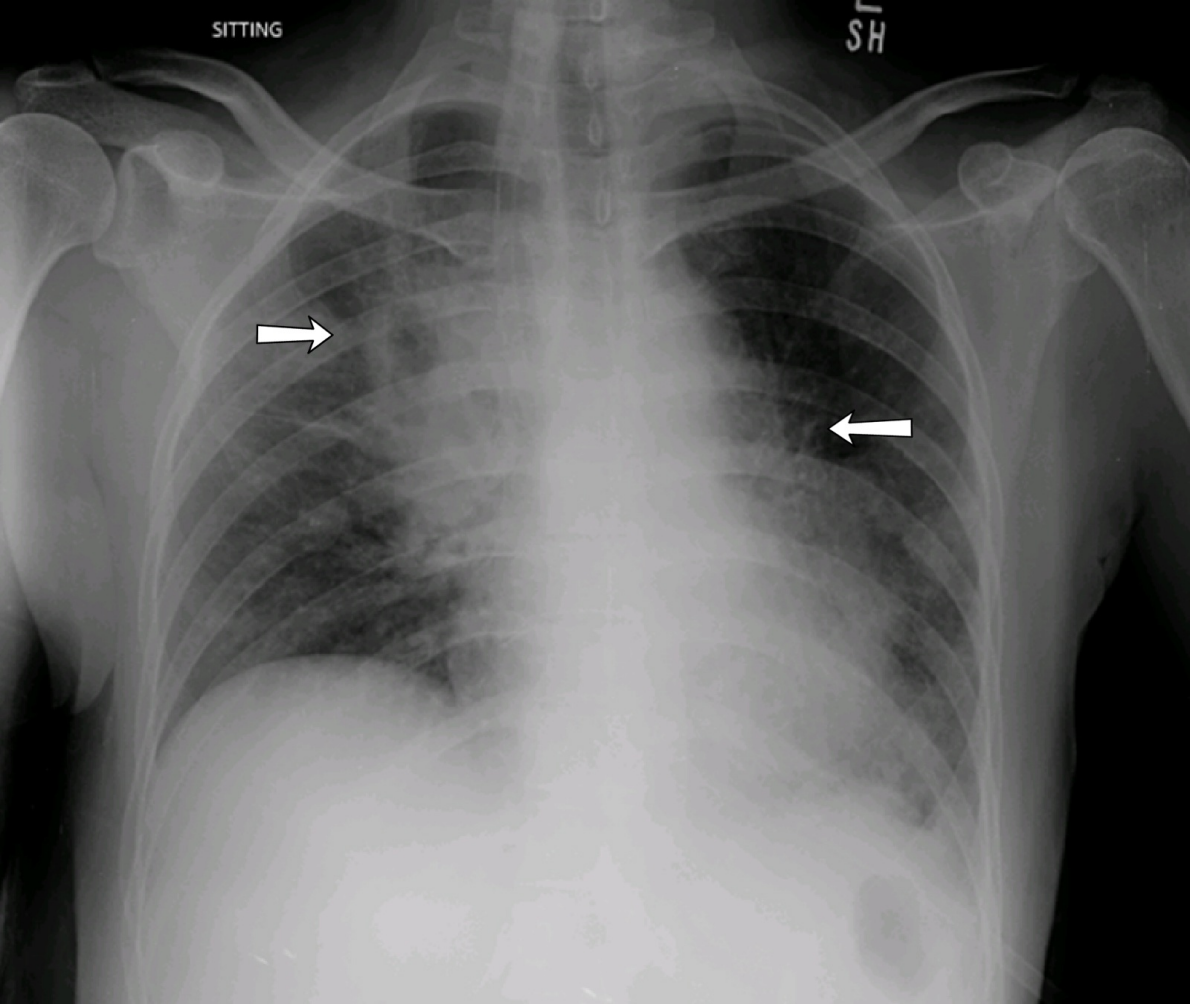
Image showing the chest X-ray. Non-homogenous shadowing with mild volume loss and opacification over the right upper heart border (white arrow).

**Figure 2:**
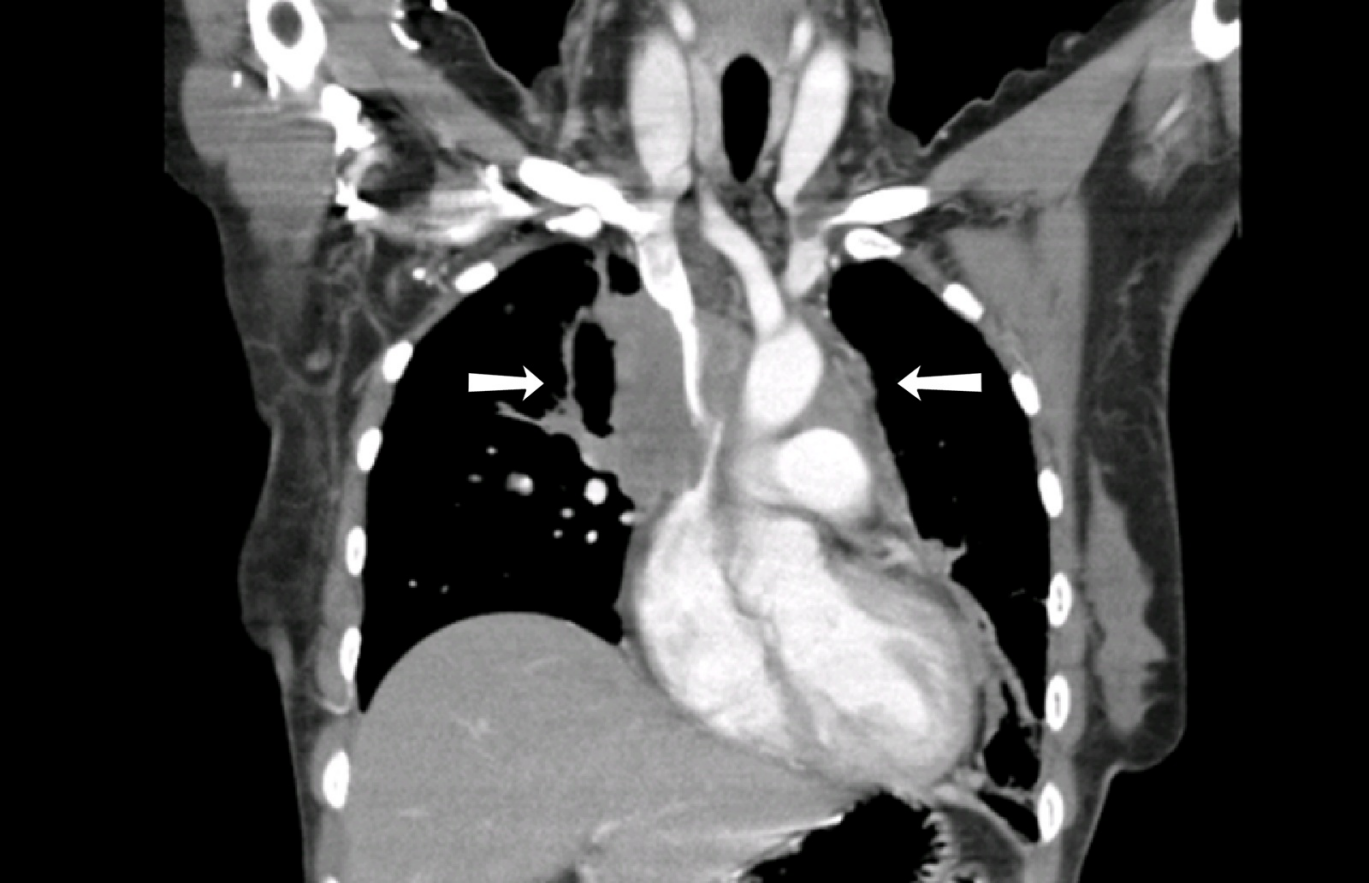
Image showing the computed tomography scan. A large ill-defined enhancing mediastinal soft tissue density mass encasing the heart and major vessels (white arrow).

**Figure 3:**
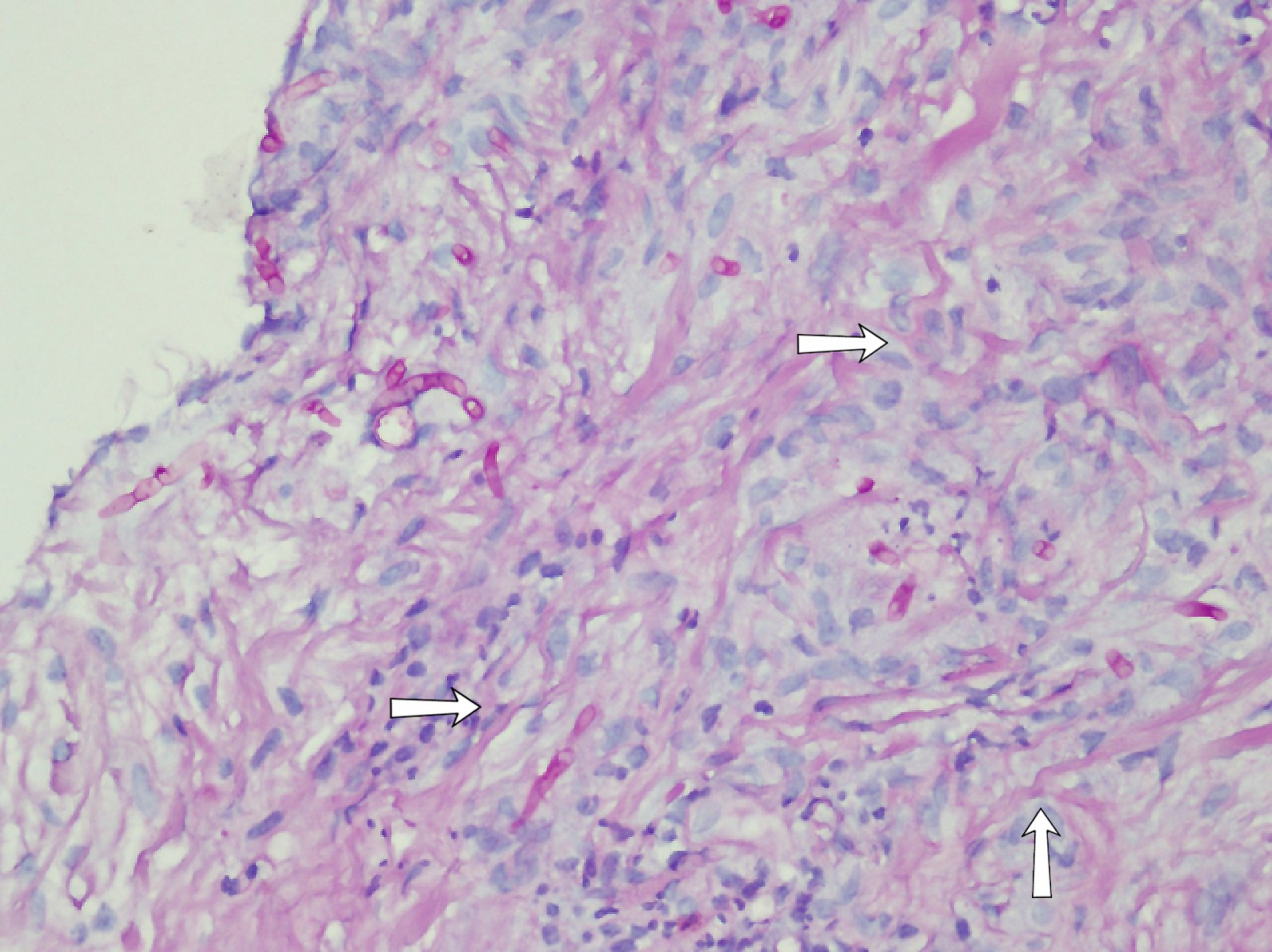
Image showing the biopsy. Chronic granulomatous inflammation with septate fungal hyphae branching at 45° angles, highly suggestive of aspergillosis (white arrow).

Basic labs were obtained that revealed a hemoglobin of 10.6 g/dL, white blood count of 18.4 g/dL with 84% neutrophils. The patients' potassium level was 3mEq/L, Lactate Dehydrogenase (LDH) was 1134 U/L and C-reactive protein (CRP) was 15 mg/dL. The rest of the laboratory results were unremarkable. A chest x-ray was obtained that showed opacification concerning for mass over the right upper heart border (Figure 1). The computed tomography (CT) scan demonstrated a large, ill-defined, enhancing mediastinal soft tissue mass (11 X 10cm) encasing the heart and major vessels, with interval increase in size as compared to a previous examination performed two months ago (9 x 6 cm). Additional findings included multiple parenchymal deposits in both lungs (Figure 2). The features raised suspicion of a lymphoma. The patient underwent a CT-guided biopsy of the mass and was hospitalized for further monitoring. The patient remained stable for two days before developing hypoxic respiratory failure along with fevers of 101-102°F. The patient demonstrated tachycardia with a heart rate of 110/min and examination was significant for stridor. The pathology report on biopsies showed a chronic granulomatous inflammation with septate fungal hyphae pointing towards invasive aspergillosis (Figure 3). An echocardiogram was obtained that showed an ejection fraction of 25-30%, severe global hypokinesia, moderate mitral regurgitation, pulmonary artery systolic pressure of 40 mmHg and an enlarged echogenic density in the right atrium. The patient was emergently transferred to the intensive care unit (ICU) and was immediately started on antifungal treatment with amphotericin and fluconazole. Unfortunately, the patient became dyspneic and hemodynamically unstable later that night. The patient was then intubated and started on three different vasopressors (epinephrine, norepinephrine, and dopamine) to keep the patients' mean arterial pressure (MAP) >65mmHg. The patient underwent cardiac arrest with pulseless electrical activity and cardiopulmonary resuscitation was carried out for 30 minutes before the code was called off.

The patients' past medical history was significant for suspected pulmonary tuberculosis with prolonged symptoms of cough and fever. The patients' Acid- fast bacilli (AFB) smears at that time were negative; however, she was treated with Anti-Tuberculosis Therapy (ATT) due to clinical suspicion. Her chest x-ray improved after completion of her therapy. The computed tomography chest obtained post therapy revealed fibrotic calcific foci with partial loss of lung parenchyma confirming the suspicion of tuberculosis. The patient then presented a few months later with chest pain and hemoptysis to another hospital and a high-resolution CT scan was performed that revealed patchy nodular pulmonary parenchymal changes in both lungs, extensive mediastinal and bilateral hilar lymphadenopathy, extensive lymphadenopathy in posterior mediastinum and possible left breast mass. A biopsy of the breast mass was performed that showed normal tissue. The patient underwent mediastinal incisional biopsy of the posterior mediastinal lymph node by a cardiothoracic surgeon and the biopsy was significant for chronic granulomatous inflammation and the patient was started on re-treatment regimen for tuberculosis. The patient again presented a month later with stridor, dysphagia, and cough. The patients' ATT was continued and she was also started on prednisolone 20 mg twice daily, the rationale of which was unknown, continued to experience symptoms of nausea, vomiting, undocumented fevers. The patients' prednisone dose was decreased to 15 mg twice daily and was then presented to our hospital after few weeks with aggravating symptoms as described above.

## Discussion

Immunocompetent hosts constantly inhale numerous conidia of Aspergillus fumigatus. Innate host mechanisms normally eliminate the conidia and aspergilloma, allergic bronchopulmonary aspergillosis and other uncommon clinical syndromes are the only infections observed in such hosts [3]. The patients can present with a wide array of symptoms such as fever, chest pain, shortness of breath, cough, and hemoptysis. Immunocompromised patients, especially patients with severe neutropenia, can have a transbronchial invasion. The subsequent invasion of the small pulmonary vessels, with hemorrhage and pulmonary infarct, results in invasive pulmonary aspergillosis [4]. It inflates from a benign aspergilloma to invasive aspergillosis, which rapidly progresses with a high mortality rate of 50%, despite treatment with proper antifungal management [5]. Rare cases with invasive aspergillosis have been reported, but only two cases involving fulminant invasive pulmonary aspergillosis in an immunocompetent host have been reported so far [6].

We determined our patient to be immunocompetent based on the medical history and clinical work up. The patient had normal blood sugar levels, an absence of neutropenia, negative human immunodeficiency virus (HIV) status and absence of malignancy.

The computed tomography scan findings showed ill-defined enhancing mediastinal soft tissue density mass encasing heart and major vessels including multiple parenchymal deposits which were suggestive of a lymphoma. The suspicion was followed up with a CT guided biopsy which did not show any evidence of malignancy. The bronchoscopy report was expedited at the physician's request. The report showed septate fungal hyphae characteristic of aspergillosis. The findings of CT scan and echocardiogram suggested direct bridging of the endobronchial lesion and a left intracardiac mass. Although the association was not proven with the utility of histopathology, the general course of events was highly suggestive. We, therefore, concluded that the endocardium in the left atrium was directly invaded by Aspergillus causing infective endocarditis. Antifungal therapy was commenced but it was too late to save the patient at that stage. In immunocompetent hosts, about 90% of blood cultures are negative adding to the diagnostic challenges of the case [7]. Galactomannan (GM) assay is another diagnostic modality which could be potentially used to diagnose invasive aspergillosis. Galactomannan is a part of Aspergillus species hyphae cell membrane and it is released when Aspergillus invades the blood vessels. While GM assay is gaining popularity in diagnosing immunocompromised hosts, it is essential to understand its utility in immunocompetent hosts. According to one study, the sensitivity, specificity, and negative predictive value (NPV) for a Bronchoalveolar Lavage with Galactomannan (BAL GM) level of ≥ 1.0 were 100%, 88.1%, and 100%, respectively. Notably, the positive predictive value (PPV) is only 42.9%, likely reflecting the low prevalence of pulmonary aspergillosis among immunocompetent patients. A combination of BAL microscopy and culture has a sensitivity and NPV similar to those of BAL GM detection but a higher specificity and PPV (92.5% and 54.6%, respectively). Moreover, a BAL GM test did not identify any cases that were not diagnosed by conventional methods like microscopy and culture. Therefore, there was no conclusive benefit of determining BAL GM levels in the diagnosis of pulmonary aspergillosis among non-immunocompromised hosts. Given the likelihood of false-positive results, a BAL GM test should not be ordered routinely in this patient population [8].

According to a World Health Organisation (WHO) report on the global burden of aspergillosis, all patients with a pulmonary insult are at risk of developing Chronic Pulmonary Aspergillosis. It can account for progressive lung destruction and the persistence of symptoms after successful anti-tuberculous treatment and can mimic smear-negative pulmonary tuberculosis [9]. Similarly, patients with a history of tuberculosis can also present as cases of invasive aspergillosis and the treating physician must be aware of the association and be able to recognize it.

## Conclusions

The rare nature, limited diagnostic options, and a high mortality rate make this type of case a diagnostic dilemma. Therefore, invasive aspergillosis should not be excluded from the differential diagnosis on the basis of immunocompetence. A holistic approach to relate to a past history of pulmonary insult and associating current symptoms with extra-pulmonary involvement such as heart in our case can help reach the diagnosis.
